# The Relationship between Cortical Blood Flow and Sub-Cortical White-Matter Health across the Adult Age Span

**DOI:** 10.1371/journal.pone.0056733

**Published:** 2013-02-21

**Authors:** J. Jean Chen, H. Diana Rosas, David H. Salat

**Affiliations:** 1 Rotman Research Institute, Baycrest Centre for Geriatric Care, University of Toronto, Toronto, Canada; 2 Athinoula A. Martinos Center for Biomedical Imaging, Massachusetts General Hospital, Harvard Medical School, Boston, Massachusetts, United States of America; 3 Department of Radiology, Massachusetts General Hospital, Harvard Medical School, Boston, Massachusetts, United States of America; 4 Department of Neurology, Massachusetts General Hospital, Harvard Medical School, Boston, Massachusetts, United States of America; 5 Neuroimaging Research for Veterans Center, VA Boston Healthcare System, Boston, Massachusetts, United States of America; Université de Montréal, Canada

## Abstract

Degeneration of cerebral white matter is commonly observed in aging, and the associated degradation in neural connectivity contributes to cognitive decline in older adults. Vascular dysfunction has been implicated as a potential mechanism for general age-related neural tissue deterioration; however, no prior study has examined the direct relationship between cortical vascular health and subcortical white-matter integrity. In this work, we aimed to determine whether blood supply to the brain is associated with microstructural integrity of connective tissue, and whether such associations are regionally specific and mainly accounted for by aging. We examined the association between cerebral blood flow (CBF) in the cortical mantle, measured using arterial spin labeling (ASL), and subcortical white-matter integrity, measured using diffusion tensor imaging (DTI), in a group of healthy adults spanning early to late adulthood. We found cortical CBF to be significantly associated with white-matter integrity throughout the brain. In addition, these associations were only partially tied to aging, as they remained even when statistically controlling for age, and when restricting the analyses to a young subset of the sample. Furthermore, vascular risk was not a prominent determinant of these effects. These findings suggest that the overall blood supply to the brain is an important indicator of white-matter health in the normal range of variations amongst adults, and that the decline in CBF with advancing age may potentially exacerbate deterioration of the connective anatomy of the brain.

## Introduction

Degeneration of the cerebral white matter is widely observed in aging, and has been associated with cognitive dysfunction [1]–[8]. Prior studies demonstrated strong associations between white-matter microstructural properties and cortical tissue health [9]–[14], especially in fibre structures connecting the affected cortical areas [11], [15], [16]. White-matter degeneration has also been associated with vascular risk factors and diseases such as stroke and hypertension [17], [18]. In particular, vascular disease can contribute to white-matter lesion formation (e.g. [19]–[22]), and reduced CBF may induce periventricular white-matter leukoaraiosis [23]. However, to date, there remains a gap of knowledge on the extent to which cortical blood supply is associated with tissue integrity in non-lesioned, normal-appearing white matter in the healthy-control population, or on whether such associations exist in the absence of vascular risk factors.

Diffusion tensor imaging (DTI) can provide measures of white-matter tissue microstructural integrity [24], [25], including fractional anisotropy (FA), and axial (AD) and radial (RD) diffusivity [26], [27]. Animal studies suggest that these parameters may be differentially sensitive to various histological properties. For example, reduced FA concomitant with increases in RD has been associated with demyelination [27], [28], while an increase in both AD and RD has been associated with Wallerian degeneration [26]. DTI-based observations of white-matter microstructural changes have consistently been associated with non-demented aging [24], [25], [29]–[36] and neurological diseases such as Alzheimer’s disease [14], [32], [37]–[41], cerebral small vessel disease [21] and amyloid angiopathy [42].

Prior studies in the area have primarily focused on the association between CBF and white-matter lesions. While an individual’s risk for cerebrovascular diseases increases with age (for review see [43]), recent studies demonstrate that cerebrovascular properties may already be tied to neuronal health in individuals with low disease risk (within the normal range of inter-individual variations). For instance, even in normotensive or mildly hypertensive individuals alone, elevated blood pressure was associated with reduced white-matter integrity [44]–[47]. It is known in the literature that vascular pathology precedes neurodegeneration in aging. Animal data reveal a decrease in vascular diameter in aging [48]. Moreover, immunohistochemical studies of periventricular veins, revealed an increase in vessel-wall thickness and vessel tortuosity in otherwise healthy white matter as part of normal aging [49]. Yet, no prior study has examined whether there is a general association between CBF and the integrity of normal-appearing white matter in healthy adults, and whether any such associations exist in the absence of vascular risk and advanced aging.

The goal of this study is to examine the relationship between cortical blood supply and the integrity of connective brain tissue in a healthy aging population. Specifically, we aimed to investigate: (1) whether cortical CBF positively correlates with sub-cortical white-matter microstructural health in normal aging, (2) whether this relationship can be largely explained by the presence of vascular risk, and (3) whether this relationship is specific to the regions exhibiting the most significant age associations in white-matter integrity. We hypothesized a strong association between cortical CBF and white-matter integrity, and one largely tied to aging and vascular risk. In contrast to prior studies that examined systemic vascular measures, we utilized quantitative CBF, a spatially specific metric of blood supply, measured using arterial-spin labeling (ASL) MRI. Moreover, we examined herein associations between CBF and tissue integrity in normal-appearing white matter as opposed to lesioned tissue. Our large healthy adult cohort allowed us good access to the age dependence of the measured effects, and our findings reveal potentially essential aspects of the premorbid maintenance of neural-tissue integrity, particularly the importance of effective blood flow regulation to brain health beyond the confines of diseases and advanced aging.

## Materials and Methods

### Participants

We studied 105 cognitively healthy participants, (46 men/59 women), aged between 23 and 88 years, and categorized as young (YA, age <40), middle-aged (MA, 40≤ age <60) and older adults (OA, age ≥60). We summarize the demographics in Table 1. The younger and middle-aged adults were recruited through the Massachusetts General Hospital and local community. Older adults were recruited through the Harvard Cooperative Program on Aging (http://www.hebrewseniorlife.org/research-harvard-coop-for-researchers), local senior centers, hospital resources and the local community. The older adults were cognitively healthy and were excluded if they had major neurologic or psychiatric illnesses. Individuals were also excluded for a variety of medical conditions including traumatic injury, cancer within the nervous system, significant substance abuse, or any other major health disorder as well as use of medications associated with substantial effects on cognitive abilities. Participants with mild forms of hypertension, hyperlipidemia, or type-2 diabetes were not excluded from this sample, but were noted for their conditions as vascular risk factors (Table 2). All diagnoses were by self report and, when available, based on quantitative laboratory test results. In addition, blood pressure was measured during the visit for each participant. All participants were cognitively healthy and scored >24 on the Mini Mental Status Exam (MMSE [50]), with the exception of one participant scoring 23. Detailed cognitive assessments are listed in Table 3. None of the participants suffered from severe depression, one reported moderate depression, and seven did not report on depression. All participants provided informed consent as required by the internal review board of our institution, and were imaged using a Siemens Trio 3 Tesla system (Erlangen, Germany) employing a 12-channel phased-array head coil for reception and body-coil for transmission. The acquisition details are summarized in later sections.

**Table 1:** Demographic information for young (YA), middle-aged (MA) and older (OA) participants.

Group	N	Age [yrs] All	Age [yrs] Men	Age [yrs] Women	Education [yrs]
YA	15 (9 M/6 F)	30.0±5.9	29.9±6.5	30.1±5.6	16.0±0.5
MA	46 (19 M/27 F)	51.9±5.8	50.5±6.2	52.9±5.4	16.5±2.8
OA	44 (18 M/26 F)	71.9±7.9	75.9±7.4	69.0±7.1	17.2±3.2

**Table 2:** Quantitative measures of vascular risk in middle-age and older adults.

	Vascular Risk	Fraction ofSubjects	Blood Pressure [mmHg]	HDL [mg/dL]	LDL [mg/dL]	Glucose [mg/dL]
**Middle-Aged**	Hypertension	2.1%				
	Hyperlipidemia	12.8%	89.4±1.1	56.4±3.4	116.0±6.1	90.5±1.7
	Type 2 Diabetes	2.1%				
	Data unavailable	4.3%	53.2%	59.6%	59.6%	59.6%
**Older Adults**	Hypertension	23.3%				
	Hyperlipidemia	32.6%	97.2±1.4	52.8±1.5	119.5±6.0	92.2±1.2
	Type 2 Diabetes	9.3%				
	Data unavailable	4.7%	60.5%	53.5%	53.5%	44.2%

Values are listed as mean ± standard error.

[*mg/dL*] = milligrams per decilitre of blood.

[*mmHg*] = millimetres mercury.

*Blood pressure*: mean-arterial blood pressure (normal range: 80∼102 [mg/dL]).

*HDL (cholesterol)*: high-density lipoprotein (normal range: >40 [mg/dL]).

*LDL (cholesterol)*: low-density lipoprotein (normal range: 100∼130 [mg/dL]).

*Glucose*: fasting plasma level (normal range: 70∼100 [mg/dL]).

**Table 3:** Health status in all participants.

Metric	Mean
**MMSE**	28.2±1.7
**Systolic Pressure [mmHg]**	128.6±17.0
**Diastolic Pressure [mmHg]**	74.3±9.0
**TRAILSA: time (s) (#errors)**	40.2±16.8 (0.286±0.535)
**TRAILSB: time (s) (#errors)**	88.8±45.1 (0.571±0.836)
**BDI**	5.62±5.75
**Data not available**	20%

MMSE: Mini-Mental Status Exam.

TRAILSA: Trail Making Test A.

TRAILSB: Trail Making Test B.

BDI: Beck Depression Inventory.

### Cerebral Blood Flow

#### MRI acquisition

CBF measurements were made using a FAIR QUIPSS II pulsed ASL (ASL) sequence [51]. The tag and control slab thicknesses were 140 mm and 340 mm, respectively, leaving 100 mm margins at either end of the imaging slab to ensure optimal inversion profile. The QUIPSS II saturation pulse was applied to a 100 mm slab inferior to the imaging region with a 10 mm gap between the adjacent edges of the saturation and imaging slabs. Flow crusher gradients were applied with a threshold of 100 cm/s. Other imaging parameters were: 64×64 in-plane matrix, 24 slices and 3.4×3.4×5 mm^3^ voxels. The ASL acquisitions each consisted of 104 frames (52 tag and 52 control), with TI_1_ = 600 ms and TI_2_ = 1600 ms, chosen to accommodate a wide range of flow rates. The scans used a repetition time (TR) of 4 s, and an echo-time (TE) of 12 ms resulting from a ¾ partial Fourier echo-planar imaging (EPI) readout. The acquisition time per slice was 42 ms. A 2D gradient-echo EPI scan (with TR set to 10 s) was used to estimate the equilibrium magnetization of arterial blood.

#### Data processing

The raw ASL time-series were motion- and drift-corrected using FSL’s FLIRT (http://fsl.fmrib.ox.ac.uk/fsl/flirt). To minimize BOLD-contamination, the control-tag difference images were calculated using surround subtraction [52]. Longitudinal (*T*_1_) relaxation due to the slice-dependent transit delay was compensated based on the per-slice acquisition time. The ASL volumes were then averaged across time and scans (2 scans/session) to maximize signal-to-noise (SNR), following which quantitative CBF maps were obtained based on the single-compartment Standard Kinetic Model [53]. The equilibrium arterial-blood magnetization was computed as the intensity in the calibration scan adjusted for longitudinal (*T*_1_) and transverse relaxation (*T*_2_^*^) differences as well as the blood-tissue water partition coefficient (λ). Typical values for proton density, labeling efficiency, λ, *T*_1_ and *T*_2_* were assumed for all grey matter based on prior literature, as described in [54], [55].

To enable surface- and ROI-based analyses, the ASL data were resampled to a 1 mm^3^ voxel size and registered to the native-space *T*_1_-weighted anatomical images using boundary-based registration [56], as described in our previous work [57], [58]. To facilitate group-analysis, the anatomical-registered ASL data were sampled onto a cortical surface atlas using spherical registration. Group-mean CBF maps were generated using non-rigid high-dimensional spherical averaging [59].

### White-Matter Microstructure

#### MRI acquisition

The diffusion-weighted images were obtained using a twice-refocused spin echo sequence [60]: 64 slices, TR/TE = 7920/83 ms, 2-mm isotropic voxels, 60 directions, b = 700 s/mm^2^, with 10 volumes at a b-value of zero.

#### Data processing

The DTI data were motion- and eddy-current corrected, and subsequently used for computing fractional anisotropy (FA), axial (AD) and radial (RD) diffusivity using the FSL Diffusion Toolbox. Voxel-wise DTI group-analyses were performed using the white-matter skeletonization procedure which is part of Tract-Based Spatial Statistics (TBSS [61]). Each subject’s FA volume was registered to the group-average white-matter skeleton (defined at an FA threshold of 0.2), and the resulting aligned images were utilized in the voxel-wise group statistics. The transformation matrices derived for the FA maps were applied to the diffusivity and *T*_2_-intensity (*b = *0) volumes for matched processing of all image volumes. The use of the TBSS skeleton allowed us to exclude regions of significant white-matter degeneration from the group analysis.

### Cortical Thickness

#### MRI acquisition

A 3D anatomical scan was acquired using multi-echo MPRAGE [62], with 1 mm isotropic resolution, TR = 2530 ms, TI = 1000 ms, TE = 1.64, 3.50, 5.36 and 7.22 ms, field of view = 256×256 mm (sagittal), matrix size = 256×256×176, bandwidth = 651 Hz/pixel and an acceleration factor = 2 (GRAPPA).

#### Data processing

Cortical thickness measures were utilized as covariates in the cortical-surface analysis to control for any potential partial-volume contamination in CBF data. Thickness assessments were performed using FreeSurfer (http://surfer.nmr.mgh.harvard.edu), whereby cortical thickness was calculated as the closest distance from the grey−/white-matter boundary to the grey/CSF (cerebrospinal fluid) boundary at each vertex on the tessellated surface, as described previously [63].

### Statistical Analysis

#### Per-voxel white-matter analyses

The DTI-derived microstructural parameters were regressed against age and CBF of all participants based on the general linear model (GLM), with age and CBF entered as continuous variables, and controlling for image intensity in the *b = *0 volumes. The statistical significance of the regression was corrected for multiple comparisons using the permutation method [64], [65], implemented through FSL’s *randomise*. The corrected statistical maps were thickened around the TBSS skeleton for visualization purposes. We also performed two types of subgroup analyses: (1) after excluding individuals with vascular risk factors (*i.e.* hypertension, hyperlipidemia and diabetes), and (2) including only younger and middle-aged adults. These analyses examined whether vascular risk or age were predominant factors contributing to the association between CBF and white-matter integrity. In addition, we controlled for the presence of vascular risk as a categorical variable in a multi-variate GLM analysis. Analyses were performed both with and without controlling for variance due to age to further assess the importance of age in the observed associations.

#### Cortical surface-based analysis using white-matter regions-of-interest

To assess the spatial specificity of the associations between white-matter microstructural integrity and CBF, we chose as “seed” the corpus callosum, a structure exhibiting a wide range of age effects along its axis. We divided the callosum into 3 sub-regions (the genu, body and splenium) to examine potential anatomical heterogeneity in the correspondence between cortical CBF and white-matter integrity in these regions-of-interest (ROIs). The association between cortical CBF and DTI parameters in each callosal segment (extracted from TBSS maps deprojected to each subject’s native-space) was computed using a GLM analysis, regressing out covariations in cortical thickness. Outliers were identified based on the standardized residuals and removed prior to the regression analyses. The statistical tests involved spatial smoothing along the cortical surface using a circularly symmetric Gaussian kernel with a full-width at half-maximum (FWHM) of 6 mm. Correction for multiple comparisons in the surface-based analyses was performed based on Random-Field Theory [66], implemented through FreeSurfer. Furthermore, we subsequently performed a similar set of analyses, substituting cortical thickness for CBF, to determine whether the observed effects were specific to CBF or generalizable to cortical morphometry.

#### ROI analysis

To compare the regional strengths of CBF- and age-associations in white matter, we performed the Steiger’s Z-Test in representative ROIs. We also used a multi-variate GLM approach to quantify the prediction power of age and CBF (both as continuous variables) in terms of microstructural integrity in specific white-matter ROIs, as summarized in Eq. (1).



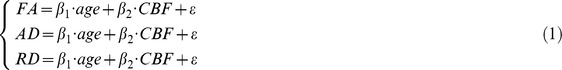
(1)


In addition, we included the DTI parameters in the GLM for each ROI, as shown in Eq. (2),



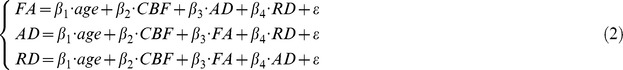
(2)with β as the regression coefficients and ε being the noise term. The GLM results corresponding to Eqs. (1) and (2) are shown in Tables 4 and 5, respectively. Finally, in select ROIs, we used two-factor analysis of variance (ANOVA) to assess potential interactions between age, CBF and white-matter DTI parameters (see Table 6). In the ANOVA, age was converted into a categorical variable (*i.e.* young, middle-aged and old), while CBF remained as a continuous variable.

**Table 4:** Multi-variate general linear modeling of white-matter structural integrity, age and CBF: Significance of fit obtained from multi-variate general linear model including individual white-matter microstructural parameters as a function of CBF and age.

Structure	Parameter	Age	CBF
Genu	FA	p<0.01*	p = 0.04*
	AD	p<0.01*	p<0.01*
	RD	p<0.01*	p = 0.03*
Body	FA	p = 0.05*	p<0.01*
	AD	*p = 0.11*	*p = 0.35*
	RD	p<0.01*	p<0.01*
Splenium	FA	*p = 0.75*	*p = 0.09*
	AD	p = 0.02*	p = 0.02*
	RD	*p = 0.62*	*p = 0.06*

The asterisk indicates statistical significance.

**Table 5:** Multi-variate general linear modeling of white-matter structural integrity, age and CBF: Significance of fit obtained from multi-variate general linear model, where each white matter microstructural parameter (i.e. FA, AD or RD) is modeled a function of CBF and age with the remaining parameters as covariates.

Structure	Parameter	Age	CBF
Genu	FA	p<0.01*	p = 0.05*
	AD	p<0.01*	p<0.01*
	RD	p<0.01*	*p = 0.18*
Body	FA	*p = 0.35*	*p = 0.19*
	AD	*p = 0.52*	*p = 0.12*
	RD	p = 0.04*	*p = 0.79*
Splenium	FA	*p = 0.28*	*p = 0.21*
	AD	p<0.01*	p = 0.01*
	RD	*p = 0.20*	*p = 0.69*

For example, the model for FA used AD and RD as covariates. The asterisk indicates statistical significance.

**Table 6:** Significance (*p*-value) obtained from two-factor ANOVA, assessing the relationship between FA, AD and RD in the callosum.

Structure	Parameter	Age	CBF	Age × CBF
	FA	p<0.01*	p = 0.01*	*p = 0.45*
Genu	AD	p<0.01*	p<0.01*	*p = 0.29*
	RD	p<0.01*	p<0.01*	*p = 0.46*
	FA	*p = 0.06*	p = 0.04*	*p = 0.85*
Body	AD	*p = 0.40*	*p = 0.08*	*p = 0.30*
	RD	p = 0.02*	p<0.01*	*p = 0.48*
	FA	*p = 0.81*	*p = 0.09*	*p = 0.53*
Splenium	AD	p<0.01*	p<0.01*	*p = 0.37*
	RD	*p = 0.50*	*p = 0.06*	*p = 0.26*

Age was used to categorize the group into young, middle-aged and older adults. The asterisk indicates statistical significance.

#### Partial-volume effects

In view of the fact that multiple tissue structures may experience volume reductions in aging, we made every effort to minimize the contribution of atrophy in our results. In the subcortical white-matter analysis, the use of the TBSS skeleton minimized potential partial-volume effects with non-fibre tissues as well as confounds due to crossing or degenerating fibres. In the surface analyses involving CBF measurements, we controlled for cortical thickness at each vertex.

## Results

### Associations between CBF and Age

The mean CBF across the cortical mantle decreased with advancing age at a rate of approximately 0.38%/year (*p*<0.05, controlled for concurrent cortical atrophy). Significant regional associations between age and CBF were found in the superior frontal and parietal, mid-inferior temporal, insular, precuneus and cingulate regions, as presented in Figure S1 (Supplementary Materials). These findings are in agreement with previously reported patterns of age-associated decline in CBF (Chen et al., 2011). All of the subjects in this prior study were involved in the current study, along with 19 additional participants.

### Associations between White-Matter Microstructure and Age

**Figure 1:**
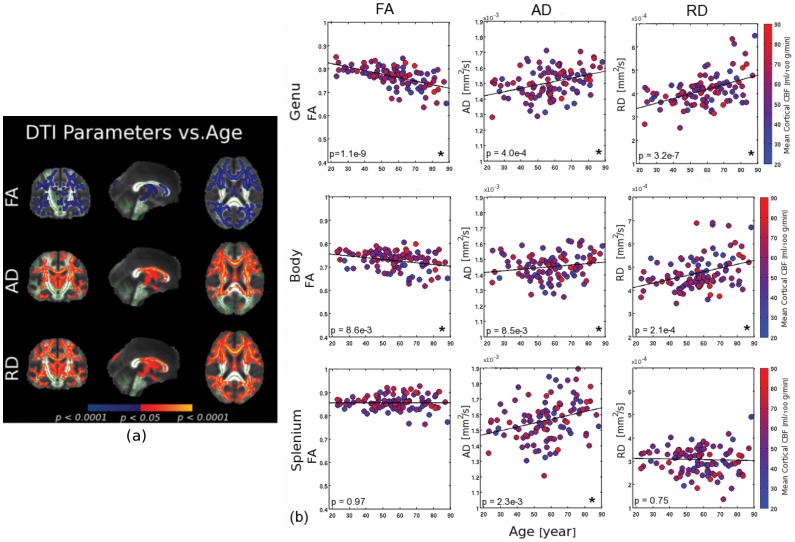
Associations between age and white-matter microstructure. (a) Aging was associated with significant decreases in fractional anisotropy (FA) (shown in blue, top panel) and increases in axial (AD) and radial diffusivity (RD) (shown in red-yellow), notably in the corpus callosum, corona radiata, cingulum, superior longitudinal fasciculus, internal capsule and uncinate fasciculus (the white matter skeleton is shown in green). (b) Relationship between age and DTI measures in the corpus callosum; the DTI measures were extracted from regions of interest defined in each participant’s native-space DTI volume. Each filled circle represents an individual subject, colour-coded for the subject’s global mean CBF. In the corpus callosum, the age-effect was most pronounced in the anterior portions. Significant age-correlations are indicated by asterisks. Note that while the CBF values show age trends (more blue to the right of the plots), there is considerable variability in CBF within age groups.

Reductions in FA were observed with advancing age (as reported in prior work), with statistically significant effects found in the corpus callosum, corona radiata, cingulum, superior longitudinal fasciculus, internal capsule and uncinate fasciculus (Figure 1A, shown in blue). FA decrease generally overlapped with diffusivity increase. Age-associated increases in axial diffusivity (AD) were more limited spatially (with fewer voxels exceeding the statistical threshold for significance), particularly in contrast to the relatively widespread increases in radial diffusivity (RD). Anterior regions of the corpus callosum exhibited the strongest statistical effects, as shown in the scatter plots in Figure 1B. We noted a considerable CBF variability in each age group. Also, the results shown in Figure 1 involve subjects with vascular risk factors.

### Associations between White-Matter Microstructure and CBF

**Figure 2:**
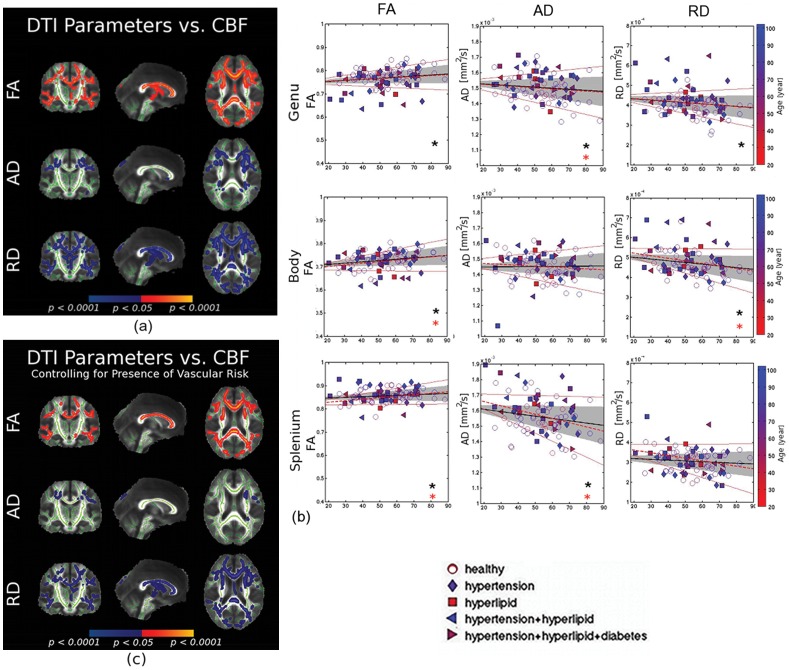
Correlation between DTI parameters and mean cortical CBF, without controlling for age. (a) The white-matter TBSS skeleton is shown in green. Cortical CBF was positively associated with white matter FA (shown in red-yellow), and negatively associated with AD and RD (shown in blue). Also, the association between DTI parameters and CBF was more evenly distributed throughout the callosum, also confirmed in the regional data plots in (b). (b) The relationship between mean cortical CBF and DTI measures in the corpus callosum, with the 95% confidence interval outlined by the shaded region. Each symbol represents one subject, colour-coded for age, with different symbols representing subjects with different vascular risk factors. The relationship between white-matter microstructure and CBF was also plotted for the risk-free subjects alone (dashed red lines), with the 95% confidence interval delimited by solid red lines. These fits show statistical similarity with the previous fits, evident from the overlapping intervals of confidence delimited by solid red lines. Significant CBF-correlations are indicated by asterisks (black for all subjects, red for risk-free subjects only). (c) Controlling for the presence of vascular risk resulted in limited changes in the observed associations between white-matter integrity and cortical CBF.

As hypothesized, cortical CBF was strongly associated with subcortical white-matter microstructural parameters (Figure 2A). In general, higher global cortical CBF was associated with higher FA as well as lower diffusivity. More specifically, AD and RD demonstrate different sensitivities, with the former being more confined to anterior white-matter regions. This spatial dependence in the association between CBF and DTI parameters was supported by the regional ANOVA results shown in Table 6.

The relationship between white-matter microstructure and CBF was also plotted for the risk-free subjects alone (dashed red lines, excluding subjects with hypertension, hyperlipidemia and diabetes. These fits show statistical similarity with the whole-group fits, evident from the overlapping intervals of confidence for the fits. However, FA in the genu and splenium of the callosum ceased to be significantly correlated with CBF once subjects with risk factors were removed (Figure 2B). Nonetheless, as confirmed at the per-voxel level in Figure 2C, after controlling for the presence of vascular risk, the association with CBF became diminished but largely unaltered for all of the DTI parameters.

**Figure 3:**
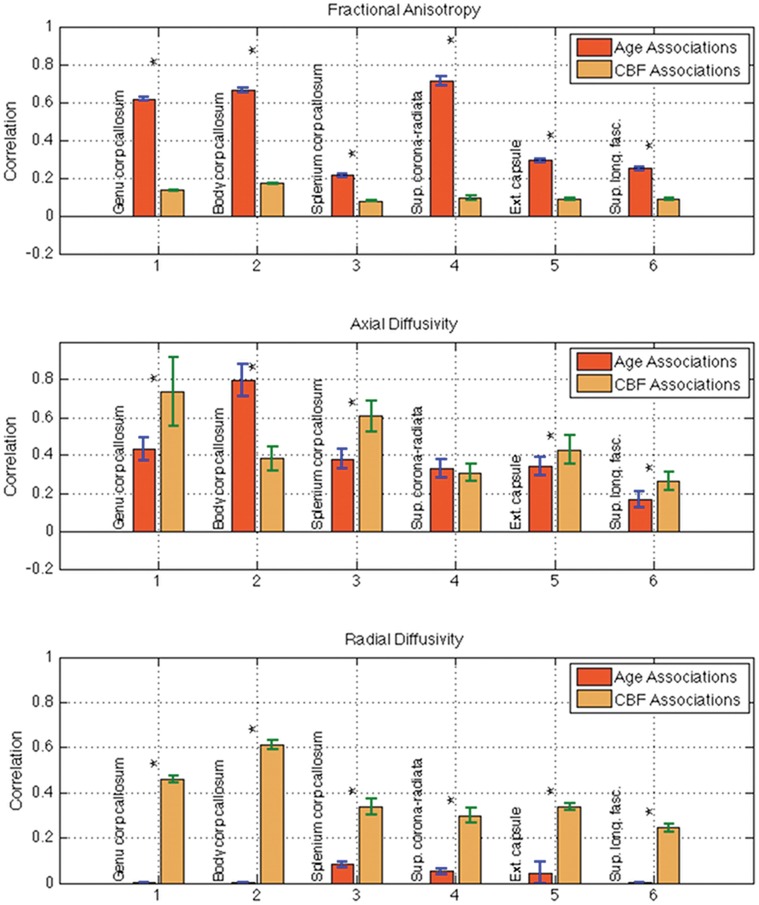
Comparisons of correlations between age/CBF and white-matter microstructural integrity in the set of six ROIs showing the most significant effects, namely the corpus callosum (genu, body and splenium), the superior corona-radiata, external capsule and superior longitudinal fasciculus. While FA was more significantly related to age, AD and RD were more significantly associated with CBF. The error bars represent the standard deviations, and asterisks indicate statistically significant differences between the age- and CBF associations.

The similarities and differences between CBF- and age-effects are quantitatively illustrated in Figure 3, in regions exhibiting the most significant associations with the two factors. The age and CBF associations are shown as regional-average correlation values, corrected for multiple comparisons. In particular, all of these ROIs exhibited more significant age-associations in terms of FA, but more significant CBF-associations in terms of RD. On the other hand, the behaviour of AD was more spatially heterogeneous. This spatial dependence in the observed effects is further demonstrated statistically through the multi-variate GLM analysis (Table 4), in which the genu of the corpus callosum was more strongly associated with both age and CBF than the splenium. This was confirmed in the multi-variate analysis involving all DTI parameters, as shown in Table 5. By including all DTI parameters in the GLM, the association between CBF and each DTI parameter becomes statistically weakened (as shown in Supplementary Figure S3) but still significant (as shown in Table 5 for the callosal ROIs).

To determine whether the observed associations between CBF and white-matter integrity were primarily due to aging, we entered age as a covariate into the GLM. Although this resulted in a reduction in the observed associations between CBF and white-matter integrity, regional associations remained strong, with substantial overlap between regions of CBF-related FA reduction and RD increases (Figure 2A). In addition, our two-way ANOVA in the callosum, which exhibits considerable heterogeneity of effects, showed no significant interaction between age and CBF in relation to the white-matter variations (see Table 6).

**Figure 4:**
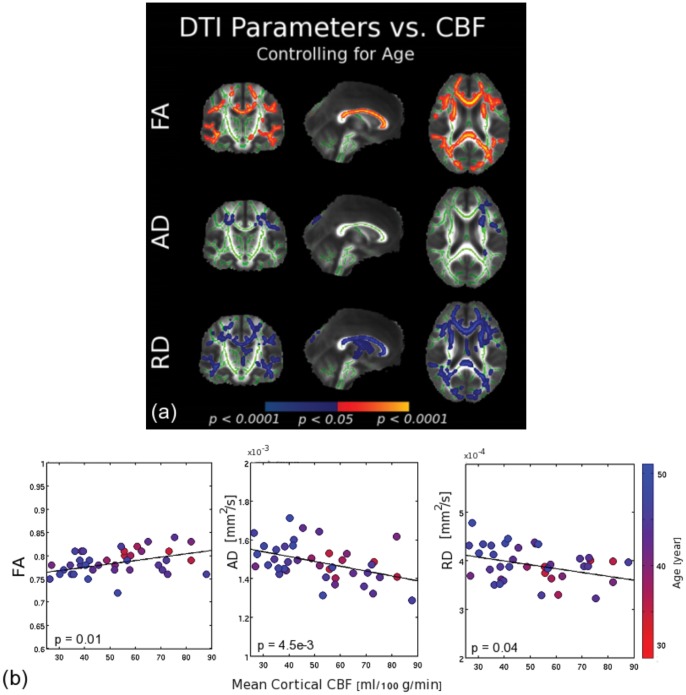
The correlation between DTI parameters and global cortical CBF, controlling for age. (a) The white-matter skeleton is shown in green. The resulting associations (positive shown in red-yellow, negative shown in blue), were well-defined and consistent with the findings when not controlling for age. There was substantial overlap between regions showing positive FA associations and negative RD associations with CBF. (b) CBF-associations in a restricted age range. As an alternate way of assessing age-independence in the CBF-DTI associations, parameters of white-matter structural integrity were significantly correlated with mean cortical CBF even when limited to an age-range between 25 and 55 years. Data is shown for the genu of the corpus callosum. Again, each symbol represents one subject, colour-coded for age.

To further explore the potential influence of age on the CBF-DTI associations, we examined this association in healthy young and middle-aged individuals alone (age = 25 to 55 years), thus avoiding the precipitous changes in white matter volume in old age. These analyses demonstrated that white-matter structural integrity is significantly correlated with mean cortical CBF even when excluding the elderly (Figure 4B), further supporting the idea that the observed associations between cortical CBF and white-matter structure are found within the normal range of inter-individual variations, and are not accounted for by age.

### Differences between the Age- and CBF-Associations with White-Matter Integrity

The statistical differences between the strengths of the CBF- and age-association with the white-matter DTI parameters are shown in Figure 3 for select ROIs, which were most strongly associated with our regressors (either age or CBF). First, it is evident that the age- and CBF-associations only partially overlap in space. While FA was more significantly correlated with age, both AD and RD were more strongly associated with CBF, more so for RD than for AD. Based on our observations in the corpus callosal ROIs, FA and RD are strongly but inversely correlated (see Figure S2 in Supplementary Materials). The two-factor ANOVA results (Table 6) showed no clear evidence of interaction between age and CBF in this context, or for a combination of the two factors to better predict DTI parameter behaviour than each factor alone.

### Spatial Specificity of the Association between White-Matter Health and CBF

**Figure 5:**
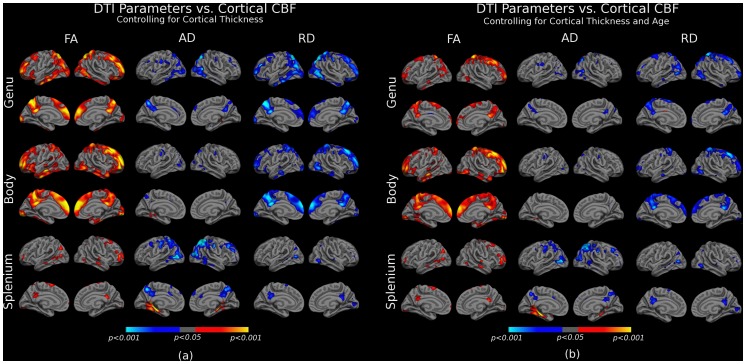
Surface-based analyses of associations between regional DTI parameters in the corpus callosum and cortical CBF. Significant positive associations are shown in orange-yellow, inverse associations are shown in blue. (a) Significant positive and spatially specific associations between FA and CBF can be seen. In contrast, AD and RD were negatively correlated with cortical CBF. (b) Strong associations remained after statistically controlling for age in the analyses. Regional associations did not follow patterns of known anatomy based on white-matter fibre trajectories or known vascular structure. However, cortical regions where CBF was associated with white matter integrity did exhibit qualitative similarities with the medial regions within the “default-mode” brain network.

To probe the anatomical specificity of the associations between DTI parameters and cortical CBF, we correlated regional DTI measures from the corpus callosum with cortical CBF maps (Figure 5). The callosum was chosen because its highly heterogeneous variations along its axis in terms of its association with age as well as CBF. Correlations between regional callosal microstructure and cortical CBF were significant and spatially selective. In general, FA was positively and diffusivity negatively correlated with regional CBF. Also, mean DTI parameters in the genu of the callosum were significantly associated with CBF in the superior frontal (lateral and medial), inferior temporal, superior lateral parietal and precuneal areas. In contrast, the body of the callosum was mainly associated with CBF in medial frontal and precuneal regions, and less so with CBF in the superior parietal region. In addition, FA and diffusivity in the splenium were for the most part not significantly correlated with cortical CBF in this analysis. Lastly, controlling for age diminished the statistical significance but did not eliminate the above associations with CBF (Figure 5B).

We performed a similar set of analyses substituting cortical thickness for CBF to determine whether the observed effects were specific to CBF or generalizable to cortical morphometry. In contrast to the CBF associations, the cortical thickness associations were predominantly accounted for by age (data not shown).

## Discussion

This study demonstrated a significant relationship between cortical blood supply (assessed via cortical CBF) and subcortical white-matter integrity (assessed via fractional anisotropy and diffusivity). Importantly, these associations were observed in healthy-appearing white matter. Moreover, these effects were not entirely explained by age or by vascular risk. In addition, these associations were spatially selective and only partially coincide with the white matter regions demonstrating the most significant age-associations. It is important to note that the current data do not permit us to ascribe causal or directional mechanisms of these associations. However, these findings provide compelling evidence for a link between cortical neurovascular physiology and white-matter health, both as a part of normal aging as well as a part of the intrinsic inter-individual variability that is not accounted for by age.

Age-associated reductions in white-matter integrity were widespread, but were more prominent anteriorly in structures such as the corpus callosum, as consistently reported in prior work [20], [30], [31], [67]. In contrast, the association between cortical CBF and white-matter integrity was more evenly distributed between anterior and posterior regions of the callosum, suggesting potentially distinct mechanisms underlying than found in aging. Our findings also raise the possibility that the measured associations reflect to a large degree physiological variability among healthy individuals, not dominated by age but instead influenced by other biological and environmental factors. In contrast, the associations between cortical thickness and white-matter microstructure were dominated by age-associated variance. Ongoing work is exploring potentially mediating factors behind these distinct observations.

Previous studies have found an association between white-matter lesion formation and risk for neurovascular diseases [68], [69]. Also, periventricular white-matter lucency has been linked to impaired cerebral autoregulation [16], potentially associated with age-related arterial stiffening and attenuation of autoregulatory capacity [70], [71]. Although such associations have been experimentally demonstrated in patients with type 1 diabetes [72], the relationship between vascular health and white-matter integrity has yet to be demonstrated in normal aging. Interestingly, we noted that while certain vascular risk factors, such as hypertension and diabetes, are associated with reduced CBF [73], [74], the inclusion of subjects with mild vascular risks did not significantly alter the trends in the DTI-CBF relationship we observed. This finding supports our view that vascular function and white-matter integrity co-vary in a healthy adult population irrespective of vascular impairment. Nonetheless, it is important to note that CBF is one of many indices of cerebrovascular health. For instance, while CBF is correlated with blood pressure [75], CBF alone does not determine vascular reactivity or neurovascular interactions. In our future work, we will examine more direct mechanisms underlying the results reported here.

While all white-matter structural parameters exhibited strong associations with age and with CBF, radial diffusivity (RD), a potential indicator of myelo-degeneration in aging [27], [28], [76], was more markedly associated with CBF (than with age) than the remaining DTI parameters (results controlled for age). This suggests that reductions in perfusion, or more generally, compromised cerebrovascular health, may contribute to white-matter damage through a “low-flow” or “low-grade” ischemic mechanism [68], [77]. This mechanism may be distinct from the primary mechanism driving age-related axial diffusivity (AD) increases, as axial diffusivity was more strongly related to age than to CBF. Also, the larger extent of RD association with CBF (when compared to AD-associations) may indicate a preponderance of myelin degeneration with advancing age. Additional work is necessary to disentangle the potential mechanistic distinctions, and to determine the link between these empirical observations and specific histopathology.

It should be noted that CBF measurements reflect both neuronal metabolic activity and vascular physiology, giving rise to two alternative interpretations to our findings. Under the metabolic hypothesis, white-matter integrity would impact neuronal function in regions projected to by the affected tracts, leading to a covariation between CBF and white-matter microstructure only in anatomically connected tissue regions. While ongoing work is examining this mechanism, we noted that the spatial patterns of the measured DTI-CBF associations did not follow the fibre structure of the affected white-matter regions, as demonstrated in the corpus callosum, the structural connectivity of which is well documented [78]. On the other hand, under a vascular hypothesis, there may be a more general association between cerebral perfusion and white-matter integrity, stemming from vascular damage associated with normal aging [48], [49]. However, associations between callosal microstructure and cortical CBF do not reflect the known vascular supply routes to the various callosal regions [79]. It is of note that the regions exhibiting significant CBF-DTI links, namely the superior-frontal, medial-frontal, temporal and precuneal regions, are also primary components of the “default-mode network”, associated with high glucose and oxygen metabolism, as well as vulnerability to degeneration in aging and in age-related diseases [80]. It is possible that the high baseline perfusion in these regions biased the detection of the associations. It is also possible that these highly metabolizing regions are most sensitive to changes in global blood flow, which impact the white-matter integrity in regions most vulnerable to vascular insult (e.g. regions supplied by deep penetrating vessels) [81]. Future work will probe associations among metabolic demands, CBF, and white-matter integrity in detail through the simultaneous measurement of hemodynamic and metabolic variables. Irrespective of the mechanism underlying the observed associations, our findings may provide important mechanistic insight into understanding the age-associated decline in connective tissue integrity [82], [83].

A recent study examining the relationship between diffusivity and CBF, both measured in the white-matter, found lower perfusion in apparently healthier fibres, attributed to their higher degree of myelination, hence lower energy demand and greater impedance for vascular penetration [84]. However, as histological studies of vascular anatomy demonstrate a continuous vascular supply path between grey and white matter [85], highly perfused white-matter should underlie highly perfused cortex in the same vascular territory. Such a scenario would predict greater white-matter integrity with higher CBF, as demonstrated here and in prior PET work [23]. These contradicting findings represent alternate models of how CBF relates to neural health, and remain to be reconciled.

The current results should be interpreted in view of the potential caveats. First, given the nature of diffusion-weighted contrast, white-matter diffusivity may potentially contain contributions from microvascular blood flow. However, within the white-matter, blood vessels are oriented along the main fibre direction [84]; hence, if the diffusivity measures had significant perfusion contribution, one would expect high CBF to correspond to high axial diffusivity, which was not the case here, precluding significant vascular contribution. Secondly, both ASL and DTI are intrinsically sensitive to subject-motion. However, motion would be expected to result in global rather than these spatially specific biases, suggesting that artifacts have limited influence. Thirdly, the older adults in the current sample contained more women than men, with the men being older than the women and blood pressure trends were slightly lower than normative values in this age range (compared with [86]). These factors may affect the generalizability of these results. The levels of education seen in our older population exceeded those of our other age groups, but not at a statistically significant level. Fourthly, while prior literature associated AD and RD to different histological features, such interpretations can be influenced by technical limitations with regard to DTI acquisition and analysis, therefore should be made with caution. Fifth, as a caveat to the interpretation, while we have shown that certain regions are more strongly associated with CBF than age, and vice versa, our study does not demonstrate a quantitative causality. It is possible, as we alluded to earlier, global cortical CBF is in part a measure of cerebrovascular health, and the most vulnerable white-matter regions would be associated with an overall decline in CBF, irrespective of the spatial distribution of the latter. Finally, we note that the reported associations were found in cross-sectional sample. The longitudinal trajectories of CBF and DTI measures is investigated in our ongoing work, and may provide information about the causality of the associations investigated here.

### Summary

Using DTI in conjunction with pulsed ASL perfusion imaging, we found a link between cortical CBF and subcortical white-matter microstructural health. The associations were regionally specific, not simply accounted for by age or by vascular risk. These findings provide support for a connection between cortical vascular physiology and subcortical white-matter health, and may have important implications for understanding the basic mechanisms of neurodegeneration.

## Supporting Information

Figure S1 **The relationship between age and cortical CBF in the studied cohort (** ***N*** **= 105).** The lateral (top) and medial (bottom) surfaces are shown for the left (L) and right (R) cortical surface. Blue indicates a negative correlation, namely, CBF becomes lower with increasing age. In order to minimize partial-volume confound, this relationship has been controlled for concurrent changes in cortical thickness.
(TIF)

Figure S2 **Correlation matrix for DTI-derived white-matter microstructural parameters.** There was no clear correlation trends between FA, AD and RD in the various white-matter ROIs. RD is strongly and negatively correlated with FA across these ROIs.
(TIFF)

Figure S3 **Associations between cortical CBF and DTI parameters of white-matter integrity, controlled for age (left), contrasted with the results of a multivariate analysis in which each DTI parameter is also modeled as a function of the remaining DTI parameters (right).** The latter method results in a much weakened association between the modeled parameter and CBF, which reflects the effect of CBF independent that is unique to the modeled DTI parameter.
(TIFF)
