# Supplementary figures and images for: The Relationship between Cortical Blood Flow and Sub-Cortical White-Matter Health across the Adult Age Span
Source: PLoS One. 2013 Feb 21;8(2):e56733. doi: 10.1371/journal.pone.0056733 (PMC3578934; doi:10.1371/journal.pone.0056733)

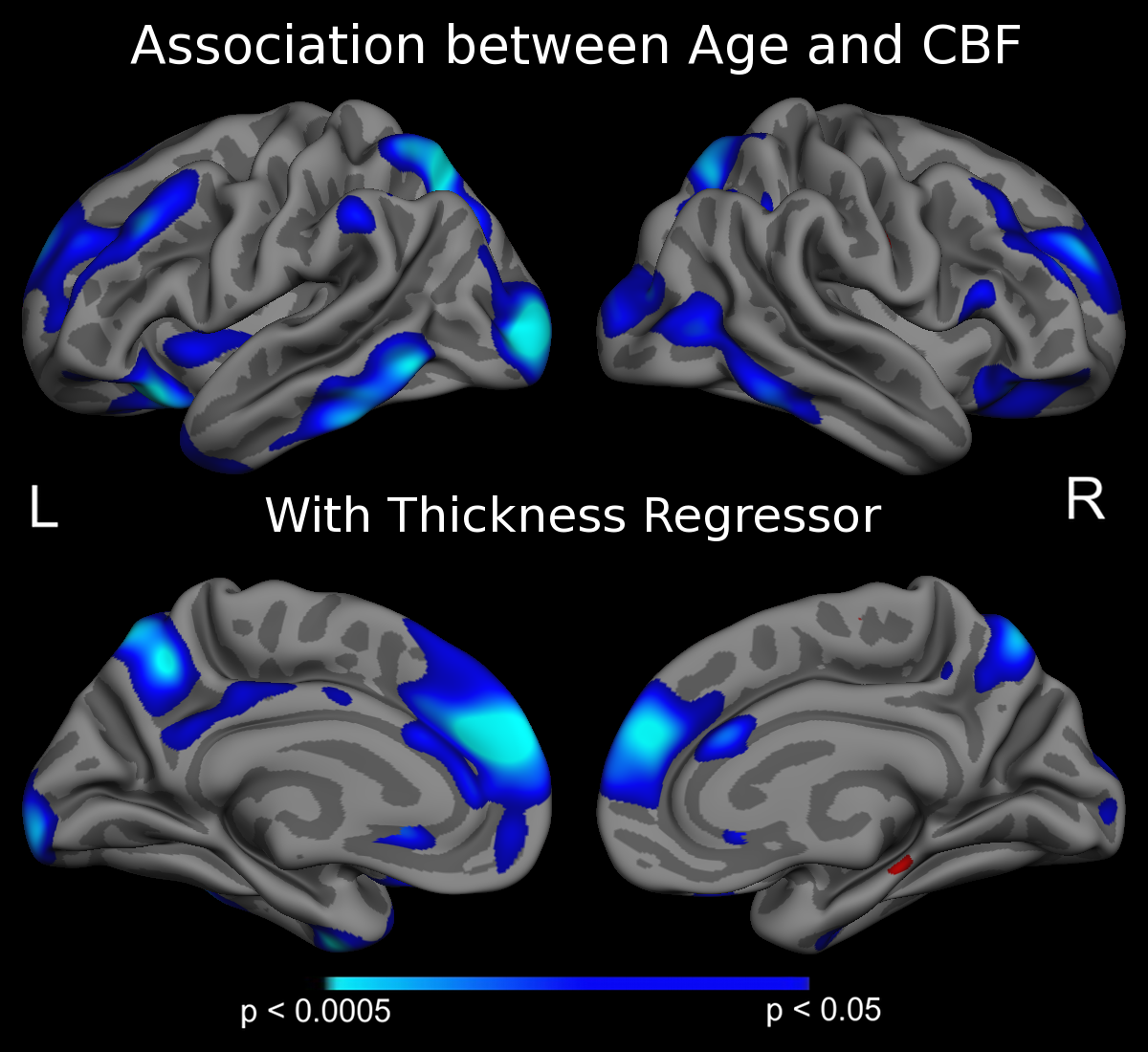

Supplement: Figure S1 — The relationship between age and cortical CBF in the studied cohort ( N = 105). The lateral (top) and medial (bottom) surfaces are shown for the left (L) and right (R) cortical surface. Blue indicates a negative correlation, namely, CBF becomes lower with increasing age. In order to minimize partial-volume confound, this relationship has been controlled for concurrent changes in cortical thickness. (TIF) [file pone.0056733.s001.tif]

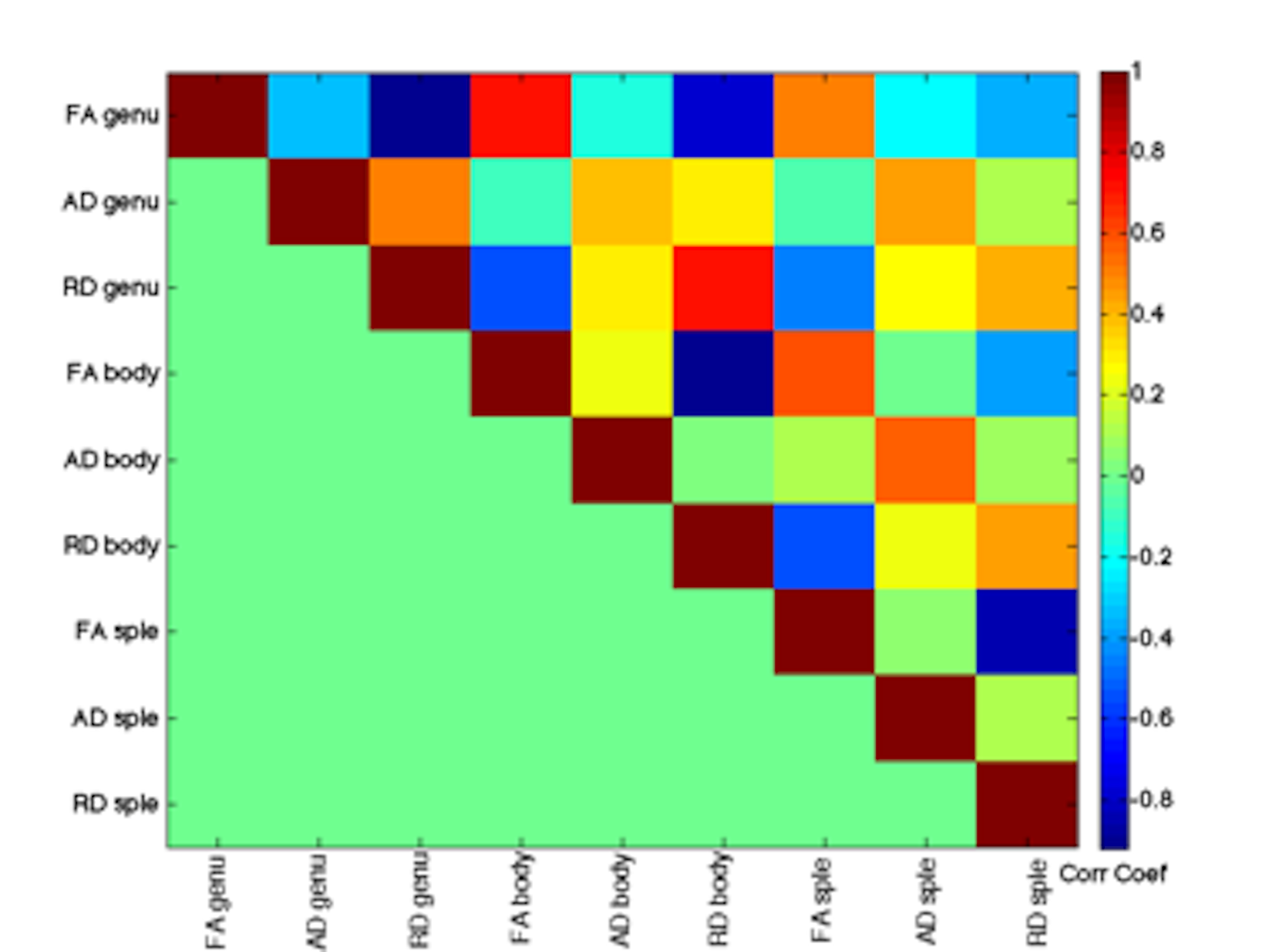

Supplement: Figure S2 — Correlation matrix for DTI-derived white-matter microstructural parameters. There was no clear correlation trends between FA, AD and RD in the various white-matter ROIs. RD is strongly and negatively correlated with FA across these ROIs. (TIFF) [file pone.0056733.s002.tiff]

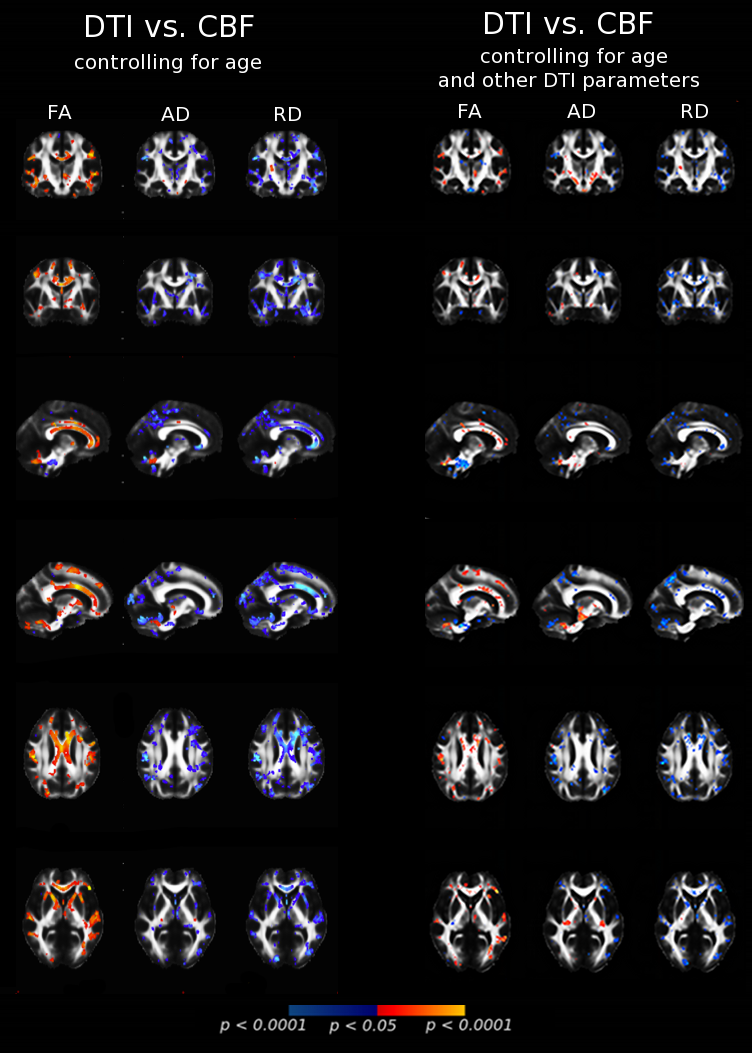

Supplement: Figure S3 — Associations between cortical CBF and DTI parameters of white-matter integrity, controlled for age (left), contrasted with the results of a multivariate analysis in which each DTI parameter is also modeled as a function of the remaining DTI parameters (right). The latter method results in a much weakened association between the modeled parameter and CBF, which reflects the effect of CBF independent that is unique to the modeled DTI parameter. (TIFF) [file pone.0056733.s003.tiff]
